# Supplementary material for: Uterine Cavity Lavage Mutation Analysis in Lithuanian Ovarian Cancer Patients
Source: Cancers (Basel). 2023 Jan 30;15(3):868. doi: 10.3390/cancers15030868 (PMC9913062; doi:10.3390/cancers15030868)
Supplement: Supplementary file 1 [file cancers-15-00868-s001.zip › cancers-2125909-supplementary.pdf]

Table S1. Detected mutations in uterine lavage and tissiue samples.

No.	Sample(s)	Gene	Exon	Coding	Amino Acid Change	Variant Effect	dbSNP
1	KN-014-lavage; KN-017-lavage; KN-030-paired; KN-033-paired; KN-036-paired; KN-037-paired; KN-046-lavage; KN-049-paired; KN-067-lavage; KN-073-paired; KN-081-paired; KN-086-lavage; KN-087-lavage; KN-089-lavage; KN-090-lavage	*BRCA1*	10	c.4035delA	p.Glu1346LysfsTer20	frameshift Deletion	rs80357711
2	KN-060-paired; KN-063-lavage; KN-079-lavage; KN-085-paired; KN-092-lavage		19	c.5266_5267insC	p.Gln1756ProfsTer74	frameshift Insertion	rs1217805587,rs80357906
3	KN-023-lavage		7	c.485_486delTG	p.Val162GlufsTer19	frameshift Deletion	rs80357708
4	KN-020-lavage		4	c.181T>G	p.Cys61Gly	missense	rs28897672
5	KN-078-paired			c.213-15A>G	p.?	unknown	rs886040903
6	KN-083-lavage		10	c.2481delA	p.Gly828AlafsTer18	frameshift Deletion	rs886040042
7	KN-019-paired; KN-052-paired; KN-093-paired	*BRCA2*	11	c.3847_3848delGT	p.Val1283LysfsTer2	frameshift Deletion	rs1229267914,rs80359405
8	KN-076-paired		8	c.658_659delGT	p.Val220IlefsTer4	frameshift Deletion	rs1131692273,rs80359604
9	KN-095-paired		17	c.7879A>T	p.Ile2627Phe	missense	rs80359014
10	KN-091-lavage		20	c.8572C>T	p.Gln2858Ter	nonsense	rs80359112
11	KN-094-paired	*TP53*	10	c.1024C>T	p.Arg342Ter	nonsense	rs730882029
12	KN-088-paired		5	c.524G>A	p.Arg175His	missense	rs28934578
13	KN-036-paired		6	c.578A>C	p.His193Pro	missense	rs786201838
14	KN-007-paired		6	c.645T>G	p.Ser215Arg	missense	rs1057520001
15	KN-007-paired		7	c.701A>G	p.Tyr234Cys	missense	rs587780073
16	KN-022-paired		7	c.710T>A	p.Met237Lys	missense	rs765848205
17	KN-052-paired		7	c.742C>T	p.Arg248Trp	missense	rs121912651
18	KN-019-paired		8	c.814G>A	p.Val272Met	missense	rs121912657
19	KN-085-paired		8	c.817C>T	p.Arg273Cys	missense	rs121913343
20	KN-084-paired		8	c.818G>A	p.Arg273His	missense	rs28934576
21	KN-072-paired		8	c.842A>G	p.Asp281Gly	missense	rs587781525
22	KN-007-paired; KN-058-lavage	*PIK3CA*	21	c.3140A>G	p.His1047Arg	missense	rs121913279
23	KN-001-lavage		21	c.3129G>A	p.Met1043Ile	missense	rs121913283
24	KN-003-lavage		10	c.1625A>T	p.Glu542Val	missense	rs1057519927
25	KN-047-lavage		2	c.112C>T	p.Arg38Cys	missense	rs749415085
26	KN-058-lavage		5	c.1030G>A	p.Val344Met	missense	rs1057519942
27	KN-001-lavage; KN-064-paired	*KRAS*	2	c.35G>T	p.Gly12Val	missense	rs121913529
28	KN-047-lavage		2	c.35G>A	p.Gly12Asp	missense	rs121913529

Table S1. Detected mutations in uterine lavage and tissiue samples.

29	KN-058-lavage		2	c.35G>C	p.Gly12Ala	missense	rs121913529
30	KN-001-lavage		5	c.389G>A	p.Arg130Gln	missense	rs121909229
31	KN-003-lavage		3	c.202T>C	p.Tyr68His	missense	rs398123317
32	KN-003-lavage	*PTEN*	5	c.388C>G	p.Arg130Gly	missense	rs121909224
33	KN-058-lavage		6	c.518G>A	p.Arg173His	missense	rs121913294
34	KN-047-lavage		4	c.210-1G>C	p.?	unknown	rs1114167621
Alterations detected only in tissue samples							
35	KN-082-tumor		4	c.372C>A	p.Cys124Ter	nonsense	
36	KN-081-tumor		5	c.524G>A	p.Arg175His	missense	rs28934578
37	KN-071-tumor		5	c.527G>A	p.Cys176Tyr	missense	rs786202962
38	KN-073-tumor		5	c.548C>G	p.Ser183Ter	nonsense	
39	KN-061-tumor		6	c.586C>T	p.Arg196Ter	nonsense	rs397516435
40	KN-033-tumor		6	c.613T>G	p.Tyr205Asp	missense	rs1057520008
41	KN-050-tumor	*TP53*	6	c.645T>G	p.Ser215Arg	missense	rs1057520001
42	KN-030-tumor; KN-049-tumor		6	c.659A>G	p.Tyr220Cys	missense	rs121912666
43	KN-078-tumor		7	c.711G>T	p.Met237Ile	missense	rs587782664
44	KN-037-tumor		7	c.742C>T	p.Arg248Trp	missense	rs121912651
45	KN-055-tumor		8	c.814G>T	p.Val272Leu	missense	rs121912657
46	KN-041-tumor		8	c.817C>T	p.Arg273Cys	missense	rs121913343
47	KN-059-tumor		8	c.892G>T	p.Glu298Ter	nonsense	rs201744589
48	KN-043-tumor		6	c.1093G>A	p.Glu365Lys	missense	rs1064793732
49	KN-071-tumor	*PIK3CA*	10	c.1637A>G	p.Gln546Arg	missense	rs397517201
50	KN-074-tumor		21	c.3140A>T	p.His1047Leu	missense	rs121913279
51	KN-043-tumor	*PTEN*	6	c.517C>T	p.Arg173Cys	missense	rs121913293
